# University of Michigan and Homœopathy

**Published:** 1868-04

**Authors:** 


### University of Michigan and Homœopathy.

One year ago the Legislature of Michigan passed a bill appropriating annually to the University the fraction of a mill on every dollar of the State assessment, tacking to it the condition that the Regents should establish a chair of Homœopathy in the Medical Department. At a recent meeting of the Board, *in secret session*, the condition was accepted, and an individual, a so-called homœopathist, was appointed. Profs. Ford, Armor and Greene promptly resigned, and the other members of the Medical Faculty will do so immediately, if they have not already. We understand that this condition was appended through the combination of a few members calling themselves homœopathists and the enemies of the appropriation, no one supposing that the Regents would accept it. After a year's delay, they have taken this action, hoping by the miserable subterfuge of locating the thing outside of Ann Arbor, to secure the money and at the same time so relieve the curse that the Faculty would endure it.—*Boston Medical Journal.*

